# Supplementary material for: Natural 2′,4-Dihydroxy-4′,6′-dimethoxy Chalcone Isolated from Chromolaena tacotana Inhibits Breast Cancer Cell Growth through Autophagy and Mitochondrial Apoptosis
Source: Plants (Basel). 2024 Feb 20;13(5):570. doi: 10.3390/plants13050570 (PMC10934494; doi:10.3390/plants13050570)
Supplement: Supplementary file 1 [file plants-13-00570-s001.zip › plants-2788487-supplementary.pdf]

SUPPLEMENTARY INFORMATION

A

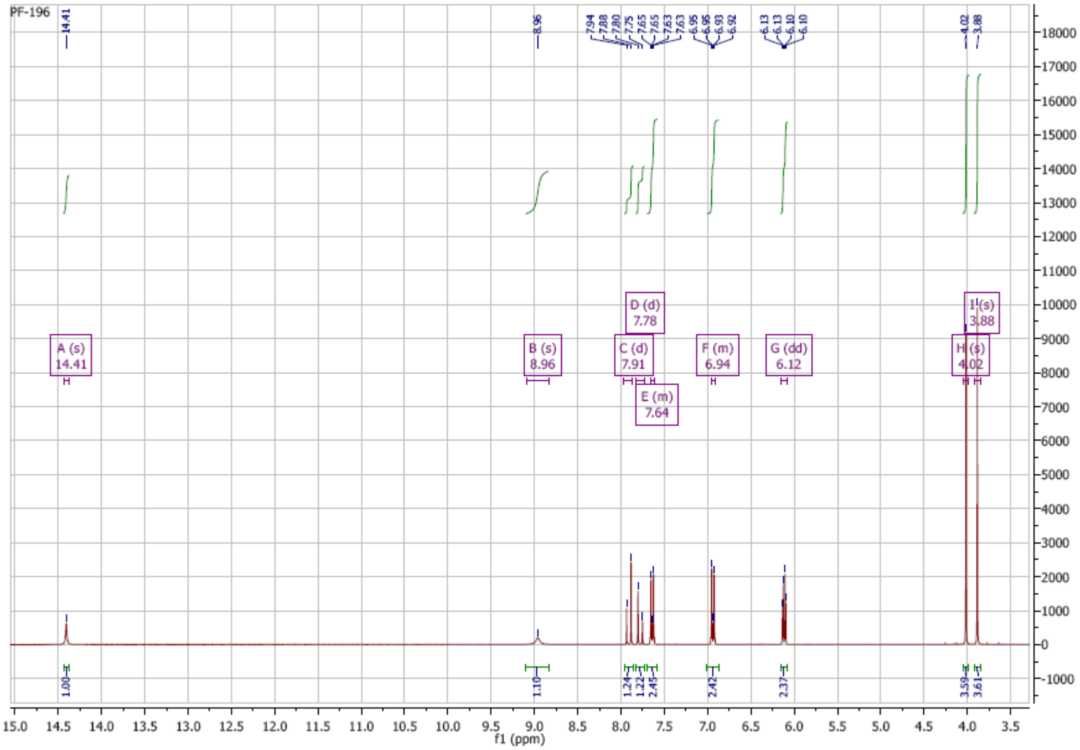

B

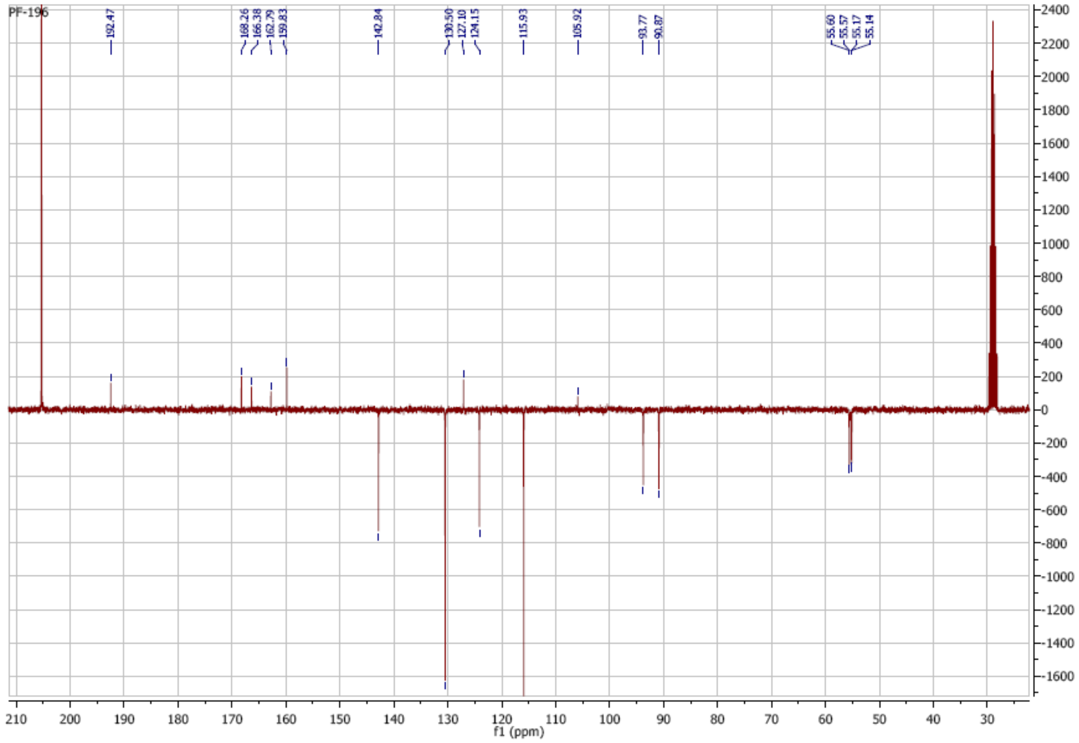

Figure S1: Nuclear Magnetic Resonance Spectroscopy of 2′4-dihidroxi-4′,6′-dimetoxichalcona: A. ^{1}H NMR and B. ^{13}C NMR spectrum.

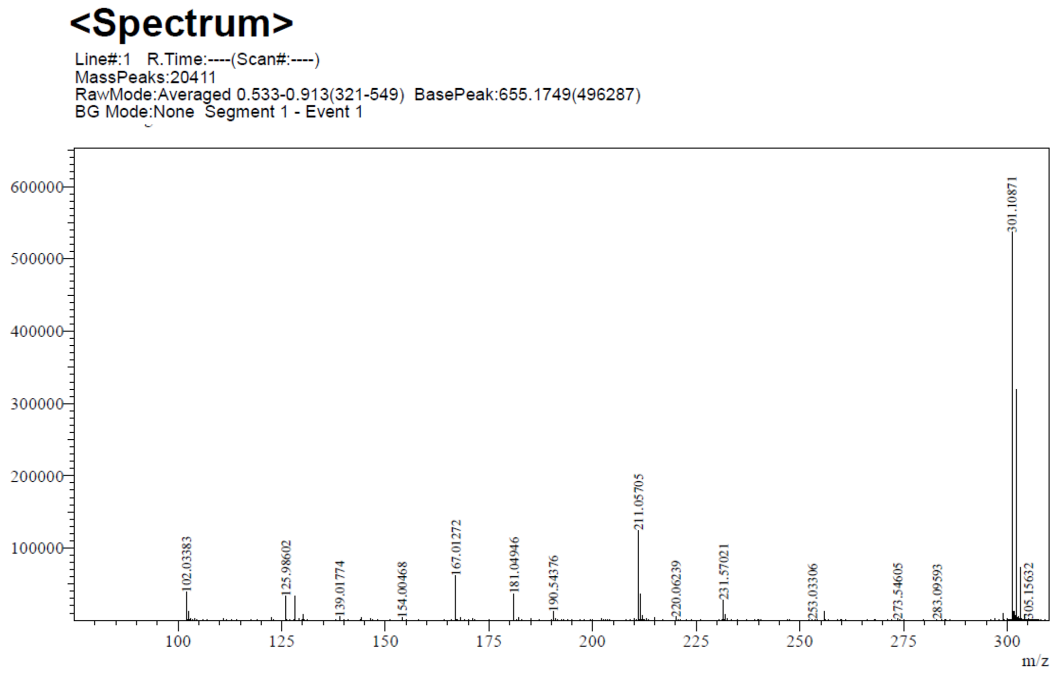

Figure S2. HPLC/QTOF analysis in the ESI positive-ion mode of 2′4-dihidroxi-4′,6′-dimetoxichalcona $[M + H]^+$: 301.10871.
